# Differences in autism features and adaptive functioning between adolescents with autism spectrum disorder and social communication disorder

**DOI:** 10.3389/fpsyt.2026.1961001

**Published:** 2026-10-02

**Authors:** Seong Hee Park, Sung Ja Cho, Naok Kang, Yoo-Sook Joung

**Affiliations:** 1 Department of Psychiatry, Samsung Medical Center, Sungkyunkwan University School of Medicine, Seoul, Republic of Korea; 2 Neudive Inc., Daegu, Republic of Korea

**Keywords:** adaptive functioning, adolescents, autism features, autism spectrum disorder, psychiatric comorbidity, social communication disorder

## Abstract

**Introduction:**

This study aimed to characterize adolescents with social communication disorder (SCD) in comparison with those with autism spectrum disorder (ASD) across two dimensions: autism features and adaptive functioning. SCD shares social communication difficulties with ASD, resulting in unclear diagnostic boundaries and raising concerns about its clinical utility given that existing research and resources remain largely ASD-oriented.

**Methods:**

Data from 76 adolescents with ASD and 44 with SCD (ages 10–18 years) were analyzed. Lifetime and current autism features were assessed using the Autism Diagnostic Interview–Revised (ADI-R) and Social Responsiveness Scale, Second Edition (SRS-2), respectively, and adaptive functioning using the Vineland Adaptive Behavior Scales, Second Edition. Propensity score matching was performed using age and Full-Scale Intelligence Quotient, and group differences were compared before and after matching. Exploratory interaction analyses additionally examined potential moderators of group differences.

**Results:**

After matching, adolescents with SCD showed less severe lifetime autism features across the core ADI-R domains, with large effect sizes (*d*s = .98–1.34, all *p* <.001). Current autism features showed smaller group differences, which remained significant across all SRS-2 subscales except Social Motivation (*d*s = .46–.68, *p* = .004–.046). Adolescents with SCD showed better adaptive functioning than those with ASD, although group differences in Communication (*p* = .085) and Socialization (*p* = .144) were no longer statistically significant after matching. Psychiatric comorbidity moderated group differences in Communication, Socialization, and overall adaptive functioning, while attention-deficit/hyperactivity disorder showed a similar but less robust pattern for Communication, which did not remain significant after correction.

**Discussion:**

Overall, accounting for age and intellectual ability, the distinction between ASD and SCD remained evident at the symptom level, whereas functional differences were partly influenced by these factors. These findings suggest that diagnostic classification alone may not fully capture the functional needs of adolescents with ASD and SCD, highlighting the importance of a multidimensional approach that considers symptom profiles, adaptive functioning, developmental and intellectual factors, and psychiatric comorbidity.

## 1 Introduction

With the transition to the Diagnostic and Statistical Manual of Mental Disorders, Fifth Edition (DSM-5), the diagnostic framework for autism spectrum disorder (ASD) was reorganized into two core domains: deficits in social communication and interaction (SCI) and the presence of restricted and repetitive behaviors (RRB) (1). This revision resulted in a substantial subset of individuals who had previously met criteria for pervasive developmental disorder not otherwise specified no longer qualifying for an ASD diagnosis. Many of these individuals exhibited marked difficulties in social communication but did not show the clinically significant level of RRB required under the new criteria (2–4). To address this diagnostic gap, a separate diagnostic category—Social communication disorder (SCD)—was introduced.

The core characteristic of SCD is a persistent impairment in the social (pragmatic) use of verbal and nonverbal communication. These difficulties may present as (a) challenges in using communication for social interaction, (b) a reduced ability to adjust language to different contexts, (c) problems adhering to conversational or narrative rules, and (d) difficulty drawing inferences from implied information. A diagnosis of SCD requires that these impairments interfere with social, academic, or occupational functioning, emerge early in development, and are not better explained by ASD, intellectual disability, or significant deficits in structural language abilities (1).

Despite its introduction in DSM-5, the conceptual boundaries of SCD remain under debate. There is no clear consensus as to whether SCD represents a diagnostic entity distinct from ASD or how the two conditions differ from one another, leaving individuals with social communication difficulties at risk of misclassification (3, 5–11). Diagnostic differentiation may further be complicated by developmental stage and intellectual ability, particularly in cognitively able individuals whose clinical features may be less clearly differentiated by conventional diagnostic measures (6–8). Sex-related variation in RRB presentation may also be relevant. Autistic females may show fewer stereotyped behaviors and restricted interests than males, while their restricted interests may appear more socially or developmentally typical; such differences may contribute to the under-recognition of ASD in females (12).

The concerns surrounding SCD are practical as much as conceptual, given that well-established treatments, clinical services, and educational support remain largely tied to an ASD diagnosis and that evidence-based interventions for SCD are few (3, 4, 6, 13). Individuals previously diagnosed with pervasive developmental disorders under DSM-IV may therefore be reclassified as having SCD under DSM-5, inadvertently limiting access to such resources (2, 4, 9, 13). These challenges highlight the need for further research to better characterize the clinical features of individuals with SCD in relation to ASD.

However, the existing literature on SCD has several limitations. First, relatively little is known about how SCD manifests in relation to ASD during adolescence, as prior research has largely focused on childhood (3, 11). Autism features and functional abilities vary with age and developmental level, as maturational processes influence both; therefore, developmental stage should be considered when assessing impairments (4, 11, 14–16). Given that adolescence is a critical developmental period marked by increasing social demands and functional expectations, examining the clinical features and real-world functional outcomes of ASD and SCD during this stage is particularly important. Second, prior research has consistently shown that intellectual ability is associated with symptom severity and may play an even greater role in shaping adaptive functioning than core symptom levels (4, 11, 17, 18). The impact of intellectual ability should therefore be carefully accounted for when assessing ASD and SCD but has not yet been adequately addressed in the existing literature (11).

In light of these gaps, the present study compared adolescents with ASD and SCD across two key domains: autism features (lifetime and current) and everyday adaptive functioning, while accounting for age and intellectual ability. We hypothesized that adolescents with SCD would exhibit less severe autism features and better adaptive functioning than those with ASD. We further hypothesized that accounting for age and intellectual ability would allow for a more precise characterization of the clinical similarities and differences between the two conditions. In addition, we explored the potential influence of individual clinical characteristics, including psychiatric comorbidity, on group differences between ASD and SCD.

## 2 Materials and methods

### 2.1 Participants

Data were obtained from two separate randomized controlled trials evaluating the efficacy of NDTx-01 (19, 20), a digital therapeutic intervention for Korean adolescents with ASD or SCD. Participants aged 10–18 years with ASD or SCD were recruited from 2023 to 2025 through the departments of child and adolescent psychiatry at five university hospitals in the Republic of Korea. Only pre-randomization baseline data from both trials, collected prior to any study intervention, were analyzed. Further details of the original studies are available in the respective trial registrations: the exploratory trial (Clinical Research Information Service, Republic of Korea; KCT0009140) and the confirmatory trial (ClinicalTrials.gov; NCT06446193).

Diagnoses of ASD or SCD were established through clinical evaluation by child and adolescent psychiatrists based on DSM-5 criteria. The exclusion criteria were as follows: (1) clinically significant behavioral or emotional dysregulation, psychotic symptoms, self-harm, or risk of harm to self or others that could interfere with study participation; (2) acute or chronic medical or psychiatric conditions requiring intensive treatment; (3) recent trauma or surgery within four weeks prior to enrollment; (4) severe neurological disorders; (5) inability to use the mobile application independently or with caregiver assistance; or (6) inability to read, comprehend, and follow study instructions.

### 2.2 Ethics approval

This secondary analysis of a combined dataset, using only anonymized baseline data, was reviewed by the Institutional Review Board of Samsung Medical Center and granted an exemption from review (SMC 2026-06-064-001). The original studies were approved as follows: the exploratory trial by the institutional review boards of Samsung Medical Center (SMC2023-05-070-011, 14 June 2023), Daegu Catholic University Medical Center (MDCR-23-008-L, 8 March 2023), and Seoul St. Mary’s Hospital (KC23DIDS0453, 27 June 2023); and the confirmatory trial by the institutional review boards of Samsung Medical Center (SMC2024-04-105), Daegu Catholic University Medical Center (MDCR-24-007-L), Seoul St. Mary’s Hospital (KC24DDDS0340), Pusan National University Yangsan Hospital (24-2024-002), and Asan Medical Center (2024-1060). Written informed consent was obtained from all participants and their caregivers in both trials, and both studies were conducted in accordance with the ethical principles of the Declaration of Helsinki.

### 2.3 Measures

#### 2.3.1 The autism diagnostic interview–revised

The ADI-R (21, 22) is a semi-structured, investigator-based interview that is widely used and well validated for assessing ASD in both clinical and research settings (23). Although not originally developed for SCD, it has been extensively utilized in studies involving both ASD and SCD populations due to overlapping social communication deficits (5, 7). The ADI-R assesses three core domains: (A) Abnormalities in reciprocal social interaction, (B) Abnormalities in communication, and (C) RRB, with diagnostic cut-offs of ≥10, ≥8, and ≥3, respectively, together with evidence of (D) Abnormality of development evident at or before 36 months of age. For each domain, two types of algorithm scores can be derived: the diagnostic algorithm, which focuses on behaviors observed at 4–5 years of age to assess lifetime autism features, and the current behavior algorithm. In this study, we used diagnostic algorithm scores as outcome measures to represent participants’ lifetime autism features, with higher scores indicating greater symptom severity.

#### 2.3.2 The social responsiveness scale, second edition

The SRS-2 (24, 25) is a parent-report questionnaire designed to measure autism symptoms observed over the past 6 months, including social communication deficits and RRB, with strong reliability and validity (26). It has also been applied in studies of SCD to evaluate social communication impairments (10). The 65-item measure yields standardized T-scores for five subscales (Social Awareness, Social Cognition, Social Communication, Social Motivation, and RRB), as well as the Social Communication Index (a composite of all subscales except RRB) and the Total score. T-scores of ≥60 indicate clinically meaningful impairment, with scores of 60–65 considered mild, 66–75 moderate, and ≥76 severe. In this study, SRS-2 T-scores were used as outcome measures to capture participants’ current autism features.

#### 2.3.3 The vineland adaptive behavior scales, second edition

The VABS-II (27, 28) is a standardized, semi-structured interview widely used to assess adaptive functioning across neurodevelopmental disorders, including ASD and SCD (15, 16, 29). For youths aged 10 years and older, it evaluates adaptive behavior in three domains (Communication, Daily living skills, and Socialization), which together yield an Adaptive Behavior Composite (ABC) score reflecting overall adaptive functioning. These are age-normed standard scores, with higher values indicating better adaptive functioning.

#### 2.3.4 The Korean Wechsler intelligence scales

The Korean Wechsler Intelligence Scales, Fourth Edition (30) were used to assess intellectual functioning. When available, previously obtained scores from age-appropriate Wechsler scales, regardless of version, were also included. The Full-Scale Intelligence Quotient (FSIQ) was used as the measure of baseline intellectual ability in the analyses.

#### 2.3.5 Psychiatric comorbidity and treatment status

Participants’ psychiatric comorbidities, including attention-deficit/hyperactivity disorder (ADHD), oppositional defiant disorder, tic disorder, depressive disorder, anxiety disorder, and obsessive-compulsive disorder, as well as lifetime treatment history and ongoing treatment status, were identified through clinical interviews and a review of medical records. Treatment referred to psychosocial interventions, including social skills training, speech-language therapy, and psychotherapy, and did not include pharmacological treatment.

### 2.4 Statistical analyses

All analyses were performed using R (version 4.5.2; R Foundation for Statistical Computing, Vienna, Austria). In the full sample, demographic and clinical characteristics were compared between the ASD and SCD groups, followed by comparisons of autism features and adaptive functioning (ADI-R, SRS-2, and VABS-II). Independent t-tests or Wilcoxon rank-sum tests were used for continuous variables, as appropriate based on their distributions, and chi-squared tests or Fisher’s exact tests were used for categorical variables. Normality was assessed using the Shapiro-Wilk test. Effect sizes were reported as Cohen’s *d*. All tests were two-tailed, with a significance level of α = .05.

Based on prior evidence (4, 17, 18, 31) that age and intelligence are important factors influencing autism features and adaptive functioning, propensity score matching (PSM) was performed using age and FSIQ as covariates to reduce potential confounding. Propensity scores were estimated using logistic regression, followed by one-to-one nearest-neighbor matching without replacement with a caliper width of 0.2 standard deviations of the logit of the propensity score. Covariate balance before and after matching was assessed using absolute standardized mean differences (ASMDs). After matching, between-group differences were re-examined in the matched sample using the same statistical methods as in the full-sample analyses.

In addition, exploratory interaction analyses were conducted to examine whether demographic and clinical factors moderated group differences across outcome measures. Separate linear regression models were fitted for each outcome, including the main effects of diagnostic group, each moderator, and the corresponding diagnostic group × covariate interaction term. The potential moderators examined were age, sex, FSIQ, psychiatric comorbidity, ADHD, lifetime treatment history, and ongoing treatment status. These analyses were conducted in the full sample to preserve statistical power and reflect clinical heterogeneity; individual comorbid diagnoses other than ADHD were not examined separately as moderators due to their small sample sizes. Type III *p*-values were used to evaluate interaction effects. Effect sizes were reported as partial η², together with unstandardized regression coefficients and 95% confidence intervals. To account for multiple testing, the Benjamini–Hochberg false discovery rate (BH-FDR) correction was applied within each measure (ADI-R, SRS-2, and VABS-II). Interaction effects with nominal *p* <.05 were subsequently examined using stratified linear regression analyses.

## 3 Results

### 3.1 Demographic and clinical characteristics

The demographic and clinical characteristics of the full sample of participants with ASD and SCD are presented in **Table 1A**. The two groups did not differ significantly in age. The sample was predominantly male in both groups (93.4% in ASD and 88.6% in SCD), with no significant difference in sex distribution. However, FSIQ was significantly higher in the SCD group than in the ASD group (91.41 vs. 82.92, *p* = .022). The SCD group had higher rates of overall psychiatric comorbidity (93.2% vs. 78.9%, *p* = .042), particularly ADHD (86.4% vs. 65.8%, *p* = .014), whereas the ASD group had higher rates of both lifetime treatment history and ongoing treatment status (65.8% vs. 40.9%, *p* = .014; and 61.8% vs. 34.1%, *p* = .006, respectively).

**Table 1:** Demographic and clinical characteristics of the ASD and SCD groups before and after propensity score matching.

A. Full sample				
Characteristic	ASD (*n* = 76)	SCD (*n* = 44)	*p*	ASMD
Age (years), Mean (*SD*)	13.24 (2.30)	12.52 (2.27)	.092	.32
Sex, male *n* (%)	71 (93.4%)	39 (88.6%)	.568	.17
FSIQ, Mean (*SD*)	82.92 (20.94)	91.41 (18.32)	.022*	.44
Psychiatric Comorbidity, Yes *n* (%)	60 (78.9%)	41 (93.2%)	.042*	.42
ADHD, Yes *n* (%)	50 (65.8%)	38 (86.4%)	.014*	.50
Tic disorder, Yes *n* (%)	2 (2.6%)	4 (9.1%)	.190	.28
Oppositional defiant disorder, Yes *n* (%)	1 (1.3%)	1 (2.3%)	1.000	.07
Depressive disorder, Yes *n* (%)	0 (0.0%)	3 (6.8%)	.047*	.38
Anxiety disorder, Yes *n* (%)	2 (2.6%)	3 (6.8%)	.355	.20
Obsessive-compulsive disorder, Yes *n* (%)	5 (6.6%)	1 (2.3%)	.413	.21
Lifetime Tx, Yes *n* (%)	50 (65.8%)	18 (40.9%)	.014*	.52
Ongoing Tx, Yes *n* (%)	47 (61.8%)	15 (34.1%)	.006*	.58

B. Matched sample				
Characteristic	ASD (*n* = 39)	SCD (*n* = 39)	*p*	ASMD
Age (years), Mean (*SD*)	13.03 (2.16)	12.69 (2.33)	.428	.15
Sex, male n (%)	36 (92.3%)	34 (87.2%)	.712	.17
FSIQ, Mean (*SD*)	92.13 (18.03)	91.05 (19.27)	.800	.06
Psychiatric Comorbidity, Yes *n* (%)	28 (71.8%)	36 (92.3%)	.036*	.56
ADHD, Yes *n* (%)	22 (56.4%)	34 (87.2%)	.006*	.73
Tic disorder, Yes *n* (%)	1 (2.6%)	4 (10.3%)	.358	.32
Oppositional defiant disorder, Yes *n* (%)	1 (2.6%)	1 (2.6%)	1.000	.00
Depressive disorder, Yes *n* (%)	0 (0.0%)	2 (5.1%)	.494	.33
Anxiety disorder, Yes *n* (%)	1 (2.6%)	2 (5.1%)	1.000	.13
Obsessive-compulsive disorder, Yes *n* (%)	1 (2.6%)	1 (2.6%)	1.000	.00
Lifetime Tx, Yes *n* (%)	24 (61.5%)	15 (38.5%)	.070	.47
Ongoing Tx, Yes *n* (%)	22 (56.4%)	12 (30.8%)	.040*	.54

ASD, Autism Spectrum Disorder; SCD, Social Communication Disorder; ASMD, Absolute Standardized Mean Difference; FSIQ, Full-scale Intelligence Quotient; ADHD, Attention-Deficit/Hyperactivity Disorder; Lifetime Tx, Lifetime treatment history; Ongoing Tx, Ongoing treatment status.

*p <.05.

PSM excluded 42 participants (37 with ASD and 5 with SCD), yielding a matched sample of 78 participants (39 per group), whose characteristics are presented in **Table 1B**. For the matching covariates, the ASMDs for age and FSIQ decreased from .32 and .44 before matching to .15 and .06 after matching, respectively, indicating improved balance following PSM. Sex also remained balanced between groups (ASMD = .17). In the matched sample, overall psychiatric comorbidity and ADHD remained more prevalent in the SCD group than in the ASD group (92.3% vs. 71.8%, *p* = .036; and 87.2% vs. 56.4%, *p* = .006, respectively). Ongoing treatment status remained more prevalent in the ASD group (56.4% vs. 30.8%, *p* = .040), whereas the difference in lifetime treatment history was no longer statistically significant (61.5% vs. 38.5%, *p* = .070).

### 3.2 Group differences in autism features and adaptive functioning in the full sample

**Table 2A** presents a comparison of autism features and adaptive functioning between the ASD and SCD groups. For lifetime autism features, the SCD group scored significantly lower across all domains of the ADI-R (largest *p* = .001). Across three core ADI-R algorithm domains (Criteria A–C), effect sizes were consistently large (*d*s = 1.24–1.54), indicating robust between-group differences in lifetime autism features.

**Table 2:** Between-group differences in autism features and adaptive functioning (ADI-R, SRS-2, VABS-II) before and after propensity score matching.

A. Full sample					
Measure	Domain/subscale	ASD (*n* = 76) mean (*SD*)	SCD (*n* = 44) mean (*SD*)	Cohen’s *d*	*p*
ADI-R	A: Abnormalities in Reciprocal social interaction	21.42 (5.87)	13.73 (6.55)	1.26	<.001*
	B: Abnormalities in Communication	14.78 (4.50)	8.09 (4.05)	1.54	<.001*
	C: RRB	6.22 (2.39)	3.34 (2.24)	1.24	<.001*
	D: Abnormality of Development evident at or before 36 months	3.53 (1.53)	2.41 (1.74)	.69	.001*
SRS-2	Social Awareness	64.51 (12.36)	58.02 (9.55)	.57	.003*
	Social Cognition	75.04 (12.40)	65.77 (11.24)	.77	<.001*
	Social Communication	81.74 (14.59)	73.39 (13.43)	.59	.002*
	Social Motivation	73.63 (15.66)	68.36 (14.12)	.35	.062
	RRB	83.70 (18.48)	71.14 (14.49)	.73	<.001*
	Social Communication Index	79.22 (14.20)	70.02 (11.29)	.70	<.001*
	Total	81.37 (14.96)	71.23 (11.60)	.73	<.001*
VABS-II	Communication	69.95 (9.66)	77.80 (11.44)	-.76	<.001*
	Daily living	74.03 (9.94)	79.34 (8.52)	-.56	.003*
	Socialization	65.53 (9.09)	70.75 (9.88)	-.56	.009*
	ABC	65.78 (8.53)	72.07 (8.46)	-.74	<.001*

B. Matched sample					
Measure	Domain/subscale	ASD (*n* = 39) mean (*SD*)	SCD (*n* = 39) mean (*SD*)	Cohen’s *d*	*p*
ADI-R	A: Abnormalities in Reciprocal social interaction	19.92 (5.47)	13.95 (6.70)	.98	<.001*
	B: Abnormalities in Communication	13.87 (4.43)	8.21 (4.02)	1.34	<.001*
	C: RRB	5.80 (2.35)	3.21 (2.13)	1.15	<.001*
	D: Abnormality of Development evident at or before 36 months	3.00 (1.58)	2.56 (1.79)	.26	.292
SRS-2	Social Awareness	64.62 (11.94)	58.08 (9.72)	.60	.010*
	Social Cognition	73.80 (11.68)	65.97 (11.31)	.68	.004*
	Social Communication	79.56 (13.74)	73.26 (13.72)	.46	.046*
	Social Motivation	72.77 (15.29)	69.18 (14.41)	.24	.289
	RRB	83.03 (19.01)	71.92 (14.27)	.66	.005*
	Social Communication Index	77.87 (14.11)	70.15 (11.70)	.60	.010*
	Total	79.90 (14.50)	71.51 (11.87)	.63	.007*
VABS-II	Communication	72.80 (7.87)	77.59 (11.53)	-.49	.085
	Daily living	75.23 (7.67)	79.77 (8.67)	-.55	.017*
	Socialization	67.21 (7.24)	70.51 (10.44)	-.37	.144
	ABC	67.69 (6.38)	72.03 (8.79)	-.56	.014*

ADI-R, Autism Diagnostic Interview–Revised; SRS-2, Social Responsiveness Scale, Second Edition; VABS-II, Vineland Adaptive Behavior Scales, Second Edition; RRB, Restricted and Repetitive Behaviors; ABC, Adaptive Behavior Composite.

*p <.05.

On the SRS-2, the SCD group exhibited significantly lower scores across all subscales (largest *p* = .003), except for Social Motivation (*d* = .35, *p* = .062). Effect sizes for the significant SRS-2 subscales were in the moderate range (*d*s = .57–.77), and were generally smaller than those for the ADI-R.

Regarding adaptive functioning measured by the VABS-II, the SCD group performed significantly better than the ASD group across all domains (largest *p* = .009). Effect sizes across the VABS-II domains were consistently in the moderate range (*d*s = .56–.76).

### 3.3 Group differences in autism features and adaptive functioning in the matched sample after propensity score matching

In the age- and FSIQ-matched sample (see **Table 2B**), adolescents with SCD showed significantly less severe lifetime autism features than those with ASD on ADI-R Criteria A, B and C (all *p* <.001), with effect sizes remaining consistently large (*d*s = .98–1.34). The only exception was ADI-R Criterion D, for which the between-group difference was no longer significant after PSM, largely due to lower scores in the ASD group. At the domain level, mean ADI-R scores in the ASD group notably exceeded the established diagnostic cut-offs across all three algorithm domains, whereas mean scores in the SCD group approximated the cut-offs, especially for Criteria B and C (Mean (SD) = 8.21 (4.02) vs. cut-off = 8, and Mean (SD) = 3.21 (2.13) vs. cut-off = 3, respectively).

Regarding the SRS-2, the pattern of current autism features remained similar before and after PSM. Social Motivation was the only subscale that did not differ significantly between the groups either before or after matching, with comparable mean scores in the matched ASD and SCD groups (72.77 vs. 69.18, *d* = .24, *p* = .289). For the other subscales, group differences remained significant after matching, with moderate effect sizes (*d*s = .46–.68), which were generally smaller than those observed for the ADI-R. At the subscale level, mean T-scores exceeded 60, indicating clinically meaningful impairment, across all subscales in the ASD group and across all but Social Awareness in the SCD group (Mean (SD) = 58.08 (9.72)).

Group differences in adaptive functioning were further attenuated after PSM. Although the SCD group continued to show higher scores than the ASD group across all domains, differences in VABS-II Communication (*p* = .085) and Socialization (*p* = .144) were no longer statistically significant. A significant group difference remained for Daily living (*d* = –.55, *p* = .017) and the ABC (*d* = –.56, *p* = .014). **Figure 1** illustrates the distribution of effect sizes across the ADI-R, SRS-2, and VABS-II before and after PSM.

**Figure 1:**
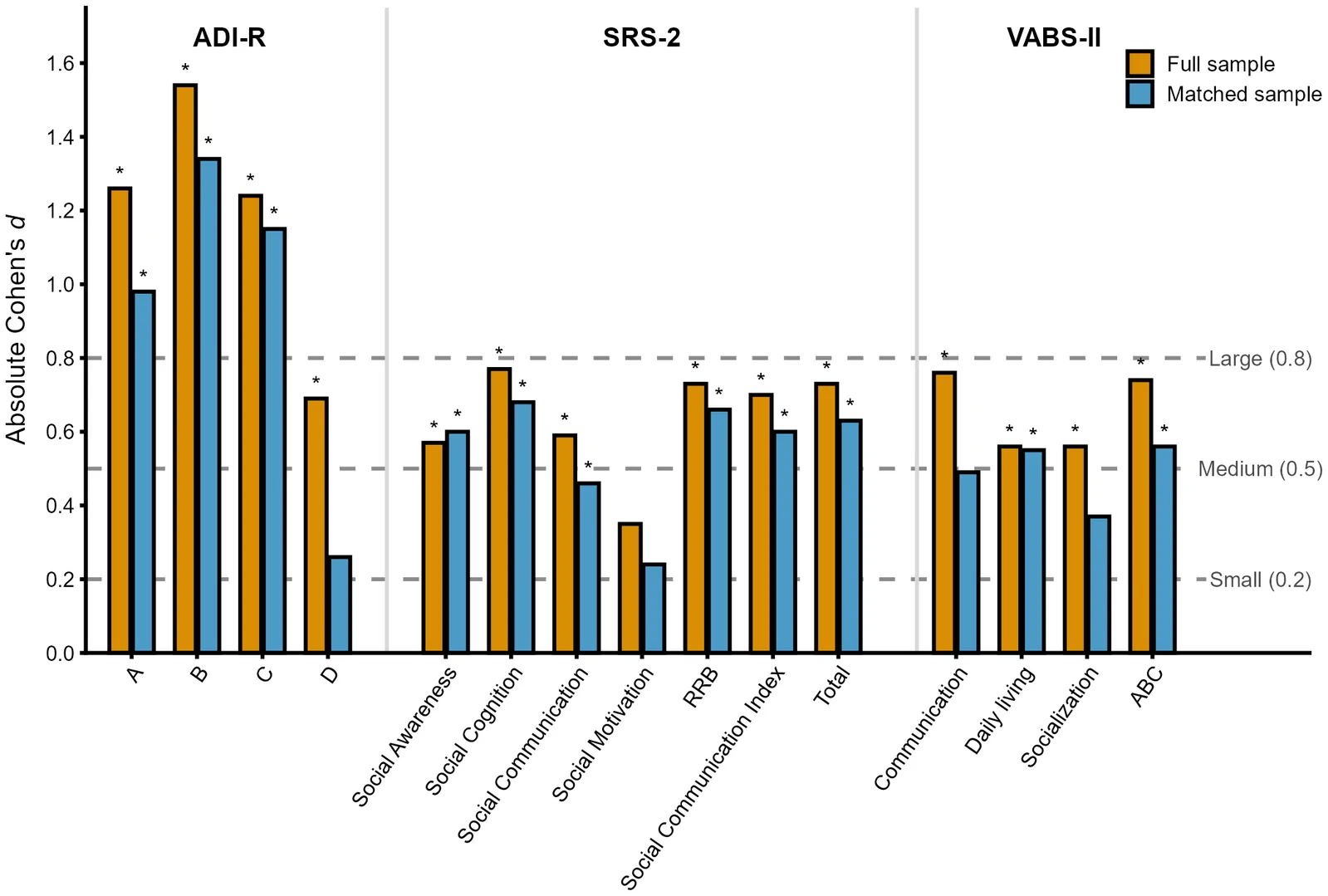
Effect sizes of between-group differences in autism features and adaptive functioning before and after propensity score matching. Absolute Cohen’s *d* values are shown for comparisons between the ASD and SCD groups in the full sample and the age- and FSIQ-matched sample. Dashed horizontal lines indicate conventional thresholds for small (*d* = 0.2), medium (*d* = 0.5), and large (*d* = 0.8) effect sizes. Asterisks indicate statistically significant between-group differences (*p* <.05). ADI-R: Autism Diagnostic Interview–Revised; **(A)** Abnormalities in Reciprocal social interaction; **(B)** Abnormalities in Communication; **(C)** Restricted and Repetitive Behaviors (RRB); **(D)** Abnormality of Development evident at or before 36 months; SRS-2, Social Responsiveness Scale, Second Edition; RRB, Restricted and Repetitive Behaviors; VABS-II, Vineland Adaptive Behavior Scales, Second Edition; ABC, Adaptive Behavior Composite; ASD, Autism Spectrum Disorder; SCD, Social Communication Disorder; FSIQ, Full-scale Intelligence Quotient.

### 3.4 Moderation analyses of group differences

In exploratory interaction analyses conducted to identify potential moderators, no significant interactions were found for the ADI-R or SRS-2 (see **Supplementary Table S1**). For the VABS-II, significant negative interactions between diagnostic group and psychiatric comorbidity were observed for Communication (B = −20.07, 95% CI [−32.88, −7.27], partial η² = .075, *p* = .003, adjusted *p* = .047), Socialization (B = −17.08, 95% CI [−28.78, −5.38], partial η² = .066, *p* = .005, adjusted *p* = .047), and ABC (B = −15.98, 95% CI [−26.56, −5.40], partial η² = .070, *p* = .004, adjusted *p* = .047), all of which remained significant after BH-FDR correction. A significant negative interaction between diagnostic group and comorbid ADHD was also observed for VABS-II Communication before correction (B = −10.11, 95% CI [−19.92, −0.29], partial η² = .034, *p* = .046) but did not remain significant after correction (adjusted *p* = .322). **Table 3** presents only the interaction effects that were significant prior to correction, and the results of all interaction tests are provided in **Supplementary Table S1**.

**Table 3:** Moderation effects of psychiatric comorbidity and ADHD on diagnostic group differences in adaptive functioning (VABS-II).

Moderator × group	VABS-II domain	B	95% CI	Partial η²	*p*	Adjusted *p*
Psychiatric comorbidity × Group	Communication	-20.07	[-32.88, -7.27]	.075	.003*	.047*
	Socialization	-17.08	[-28.78, -5.38]	.066	.005*	.047*
	ABC	-15.98	[-26.56, -5.40]	.070	.004*	.047*
ADHD × Group	Communication	-10.11	[-19.92, -0.29]	.034	.046*	.322

Only interaction effects with *p* <.05 before Benjamini–Hochberg false discovery rate (BH-FDR) correction are presented. Adjusted *p*-values were calculated using the BH-FDR correction.

VABS-II, Vineland Adaptive Behavior Scales, Second Edition; ABC, Adaptive Behavior Composite; CI, confidence interval; ADHD, Attention-Deficit/Hyperactivity Disorder. B = unstandardized coefficient for the Moderator × Group interaction term.

*p <.05.

### 3.5 Stratified analyses of interaction effects

Stratified analyses further examined these exploratory interaction patterns (see **Table 4**). When stratified by psychiatric comorbidity, group differences were substantially larger among participants without comorbidity. Among individuals without psychiatric comorbidity, the SCD group outperformed the ASD group on VABS-II Communication (B = 26.73, 95% CI [16.70, 36.75], *p* <.001), Socialization (B = 21.29, 95% CI [10.37, 32.22], *p* = .001), and the ABC (B = 21.29, 95% CI [11.88, 30.70], *p* <.001). In contrast, among participants with psychiatric comorbidity, between-group differences were much smaller (Communication: B = 6.66, 95% CI [2.63, 10.69], *p* = .002; Socialization: B = 4.21, 95% CI [0.62, 7.81], *p* = .024; ABC: B = 5.31, 95% CI [2.03, 8.59], *p* = .002). A similar pattern was observed for ADHD, with a larger between-group difference in Communication among participants without ADHD (B = 17.21, 95% CI [7.85, 26.56], *p* = .001) than among those with ADHD (B = 7.10, 95% CI [2.97, 11.23], *p* = .001). However, given that the interaction between diagnostic group and comorbid ADHD did not remain significant for Communication after correction, the corresponding stratified findings should be considered exploratory and interpreted with caution.

**Table 4:** Diagnostic group differences in adaptive functioning (VABS-II), stratified by psychiatric comorbidity and ADHD status.

VABS-II domain	Stratum	B	95% CI	*p*
Communication	Overall	7.85	[4.01, 11.69]	<.001*
	Psychiatric comorbidity [Yes]	6.66	[2.63, 10.69]	.002*
	Psychiatric comorbidity [No]	26.73	[16.70, 36.75]	<.001*
Socialization	Overall	5.22	[1.74, 8.71]	.004*
	Psychiatric comorbidity [Yes]	4.21	[0.62, 7.81]	.024*
	Psychiatric comorbidity [No]	21.29	[10.37, 32.22]	.001*
ABC	Overall	6.29	[3.14, 9.45]	<.001*
	Psychiatric comorbidity [Yes]	5.31	[2.03, 8.59]	.002*
	Psychiatric comorbidity [No]	21.29	[11.88, 30.70]	<.001*
Communication	Overall	7.85	[4.01, 11.69]	<.001*
	ADHD [Yes]	7.10	[2.97, 11.23]	.001*
	ADHD [No]	17.21	[7.85, 26.56]	.001*

ADHD-stratified findings should be considered exploratory because the ADHD × group interaction did not remain significant after BH-FDR correction.

VABS-II, Vineland Adaptive Behavior Scales, Second Edition; CI, confidence interval; ABC, Adaptive Behavior Composite; ADHD, Attention-Deficit/Hyperactivity Disorder. B = unstandardized coefficient for SCD relative to ASD.

*p <.05.

## 4 Discussion

This is one of the few studies comparing the clinical characteristics of adolescents with ASD and SCD and, to our knowledge, the first to directly compare symptom- and function-level outcomes after accounting for age and cognitive ability. The main findings can be summarized as follows. First, adolescents with SCD exhibited consistently less severe impairments than those with ASD across both lifetime (ADI-R) and current (SRS-2) measures, and these differences remained evident after matching for age and FSIQ. Second, although adaptive functioning was generally better in the SCD group, differences in Communication and Socialization were no longer statistically significant after matching for age and FSIQ. Last, psychiatric comorbidity moderated between-group differences in adaptive functioning, while ADHD showed a similar but less robust moderating pattern after correction.

Lifetime autism features remained significantly less severe in adolescents with SCD than in those with ASD across three core ADI-R domains (Criteria A–C), even after matching. More specifically, mean ADI-R scores in the ASD group clearly exceeded the established diagnostic cut-offs across all three core algorithm domains, whereas those in the SCD group approximated the cut-offs. This pattern reflects the diagnostic challenges surrounding SCD, as individuals with SCD exhibit distinctly less severe symptoms than those with ASD across both SCI and RRB domains, yet may still show clinical impairments that lie near the diagnostic boundary.

These findings are consistent with previous studies demonstrating a gradient of autistic symptoms across both SCI and RRB domains, with greater severity in ASD than in SCD or pragmatic language impairment, and greater severity in SCD than in typically developing individuals (2, 5, 6, 9, 10). Importantly, our findings extend previous work by directly comparing ASD and SCD after accounting for both age and intellectual ability, factors known to influence the manifestation and assessment of autism symptoms (5, 10, 11). Because previous studies have rarely accounted for both simultaneously, the persistence of large between-group differences after PSM further supports the diagnostic distinction between the two disorders at the symptom level.

As with lifetime autism features on the ADI-R, adolescents with SCD showed less severe current impairments than those with ASD across both SCI and RRB domains, before and after matching. Between-group differences were statistically significant across most SRS-2 subscales; however, effect sizes were generally moderate, suggesting relatively modest differences in current autism features. At the subscale level, mean scores in the ASD group were within the clinically elevated range across all subscales, while those in the SCD group remained elevated but generally lower across most subscales, with the mean Social Awareness score below the clinical threshold.

The only SRS-2 subscale that did not differ significantly between groups was Social Motivation. Social motivation is recognized as an important, multifaceted component of social communication in ASD and has been associated with broader clinical outcomes, including anxiety, depression, social anxiety, and everyday social functioning (32–34). However, social motivation has received relatively little attention in SCD, and direct comparisons with ASD remain scarce. Given the comparable levels of impairment observed in adolescents with SCD and ASD, social motivation may be less discriminative between the two conditions than other dimensions of autism features during adolescence. Social motivation may represent a relevant but understudied aspect of SCD and warrants further attention in research and clinical practice.

Notably, compared with the lifetime autism features assessed using the ADI-R, between-group differences in current autism features measured by the SRS-2 were generally smaller across both SCI and RRB domains. This pattern may reflect less differentiation in current symptom severity during adolescence, developmental changes in symptom expression, or both; however, these possibilities cannot be clearly distinguished given the present cross-sectional design and differences between the two measures. Although little is known about how the clinical distinction between ASD and SCD changes across development, considering this pattern within a developmental context may still be informative, given that autism features may evolve and manifest differently across development (4, 11). Relatedly, Norbury suggested that group differences between ASD and SCD observed during childhood may become less apparent during adolescence (8), offering one possible developmental interpretation of the observed pattern. Further direct comparative and longitudinal studies are needed to clarify this issue.

Adaptive functioning is an important clinical indicator of real-world functioning in individuals with ASD and has consistently been associated with long-term outcomes such as educational attainment, employment, and independent living (18). Although adaptive functioning is associated with autism symptom severity, longitudinal studies have demonstrated that it follows a partially independent developmental trajectory, supporting its role as a distinct dimension of clinical functioning (35, 36). Accordingly, we evaluated adaptive functioning alongside autism features to provide a more comprehensive comparison between ASD and SCD.

In the present study, adolescents with both ASD and SCD demonstrated a similar adaptive functioning pattern, with the greatest impairment in Socialization, followed by Communication and Daily living. This pattern is consistent with the characteristic adaptive functioning profile reported in previous studies of ASD, with Socialization representing the most impaired domain and Daily living being relatively preserved (18, 37, 38). Importantly, adaptive functioning in the SCD group also remained below age-expected levels across all domains, indicating that both ASD and SCD are characterized by functional difficulties relative to typically developing peers.

When directly comparing the two groups, adolescents with SCD demonstrated better adaptive functioning than those with ASD across all adaptive domains, with the largest group differences observed in Communication, consistent with the findings of Jain et al. (15). However, these differences were markedly attenuated after matching for age and intellectual ability, with no significant between-group differences remaining in Communication or Socialization, while the difference in daily living and overall adaptive functioning remained significant. These findings suggest that age and intellectual ability may partly account for some of the functional differences between ASD and SCD, although they do not fully explain the overall difference in adaptive functioning. This interpretation may be particularly relevant to Communication, which has shown the most consistent associations with intellectual ability among the Vineland domains (16, 18, 39), while Socialization has shown weaker associations with IQ but stronger associations with age (18, 39).

Adaptive functioning is a multidetermined construct influenced by factors including age, intellectual ability, autism symptom severity, and executive functioning (17, 18, 36, 37, 40). In ASD, adaptive functioning may fall below the level expected from intellectual ability, particularly among individuals with higher IQ (18, 31, 39, 41). Social-communication difficulties may also contribute to this discrepancy: greater social-communication symptom severity has been associated with larger IQ–adaptive functioning gaps, while childhood pragmatic language abilities predict later adaptive functioning beyond structural language abilities (31, 42). These findings suggest that everyday functioning depends not only on intellectual capacity itself but also on the ability to apply that capacity effectively across social and independent living contexts.

This discrepancy may become more pronounced during adolescence, as adaptive deficits relative to intellectual ability become increasingly apparent with age in ASD (31, 37, 41). As social and environmental demands and expectations for independent functioning increase during adolescence, individuals are required to draw on pragmatic communication and executive skills, as well as cognitive capacities, in increasingly complex and flexible ways (4, 11, 37). These increasing demands may further challenge the application of underlying capacity in everyday contexts.

Although direct evidence regarding the IQ–adaptive functioning gap in adolescents with SCD remains limited, this framework may be relevant given the social and pragmatic communication difficulties shared by SCD and ASD. Moreover, emerging transdiagnostic evidence is consistent with a role for social-communication difficulties in cognitive–adaptive functioning discrepancies across neurodevelopmental conditions (43). Together, the attenuation of Communication and Socialization differences after matching raises the possibility that, despite milder autism-related symptoms and relatively preserved intellectual ability, adolescents with SCD may also experience substantial difficulties translating cognitive potential into everyday functioning in these adaptive domains.

Our findings suggest that psychiatric comorbidity may be an important contributor to adaptive functioning. In this study, the presence of psychiatric comorbidity significantly moderated the relationship between diagnostic group and adaptive functioning, with differences particularly attenuated in Communication, Socialization, and ABC domains. Specifically, ADHD comorbidity showed a similar moderating pattern for Communication, although this interaction did not remain significant after correction for multiple comparisons.

This exploratory finding is clinically relevant given the known overlap between ADHD symptoms and pragmatic communication impairments. ADHD has been closely associated with difficulties in conversational processes, including turn-taking, topic maintenance, and the selection of relevant information (11, 38). Because pragmatic communication difficulties are central to both SCD and ASD, ADHD-related difficulties in this domain may compound the communicative vulnerabilities in both groups. Although evidence in SCD remains limited, longitudinal studies in ASD have shown that ADHD symptoms independently predict poorer adaptive functioning even after accounting for intellectual functioning and autism symptom severity (36), and pragmatic language difficulties are also associated with poorer adaptive outcomes over time (42). Together, this evidence provides a possible context for the observed moderating pattern. However, this interpretation should remain hypothesis-generating rather than confirmatory, given the limitations of our sample and exploratory analyses. Future studies with larger, more representative samples should test whether co-occurring symptoms, particularly ADHD symptoms given their clinical overlap with pragmatic communication impairments, moderate differences in adaptive functioning between ASD and SCD.

### 4.1 Clinical implications

The present findings have several clinical implications. By evaluating lifetime and current autism features together with adaptive functioning, we provide a more comprehensive characterization of the clinical profiles of adolescents with both ASD and SCD.

Given the ongoing uncertainty surrounding the clinical boundaries of SCD, the persistence of group differences in lifetime autism features after accounting for age and intellectual ability supports a clinical distinction between ASD and SCD at the symptom level, although the present findings are not sufficient to determine whether these differences reflect categorical distinctions or quantitative variation along a continuum. This finding highlights the clinical utility of a detailed developmental history, including careful assessment of RRB, when distinguishing ASD from SCD. Additionally, potential sex-related variation, particularly in RRB presentation, and its influence on clinical presentation may also warrant consideration in this assessment, although the role of sex could not be fully examined in the present study.

During adolescence, group differences in current autism features also remained evident across both SCI and RRB domains. However, these differences were less pronounced than those in lifetime features, with the lack of differentiation in Social Motivation between adolescents with ASD and SCD being of particular interest. These patterns suggest that current symptom presentations provide clinically relevant information but are better interpreted in conjunction with developmental history rather than in isolation.

Beyond these symptom-level distinctions, diagnostic classification alone did not fully capture the functional needs of affected adolescents. The attenuation of differences in adaptive Communication and Socialization after accounting for age and intellectual ability highlights the importance of individualized assessment of adaptive functioning, as an independent clinical outcome and a direct target of intervention. Comprehensive clinical assessment should therefore integrate symptom presentation and adaptive functioning with developmental and cognitive characteristics, as well as co-occurring psychiatric conditions, including ADHD. As evidence-based assessment and intervention strategies remain largely ASD-oriented, these findings may also inform the development and adaptation of clinical approaches for adolescents with SCD.

### 4.2 Limitations

Our study has several limitations. First, the cross-sectional design precluded evaluation of developmental trajectories in ASD and SCD. In addition, differences between the ADI-R and SRS-2 may partly reflect their different targets and time frames, capturing lifetime and current autism features, respectively. Longitudinal studies using comparable measures are needed to clarify how autism features and adaptive functioning change across development. Heterogeneity in Wechsler scale versions and the timing of cognitive assessments may have introduced variability in FSIQ measurement.

Second, the modest sample size may have limited statistical power and increased the risk of Type II errors; therefore, nonsignificant findings should be interpreted cautiously. Although sex distribution was balanced between the groups and sex did not significantly moderate group differences in our analyses, both diagnostic groups were predominantly male, limiting the evaluation of sex-specific patterns. Given that sex-related variation may influence symptom presentation, adaptive functioning, and clinical differentiation between ASD and SCD, further studies with larger samples and greater female representation are warranted.

The exploratory interaction analyses should also be interpreted in light of the sample characteristics, including the imbalance in psychiatric comorbidity between groups and the high prevalence of ADHD in the SCD group, which may limit the stability and generalizability of these findings. Given the exploratory nature of these analyses and the number of tests performed, BH-FDR correction was applied; the comorbidity interactions remained significant whereas the ADHD interaction did not. Accordingly, the moderation findings should be interpreted cautiously, particularly those regarding ADHD.

Finally, while psychosocial interventions were considered, concurrent psychotropic medication use was not included in the analysis, which may have introduced residual confounding. Although pharmacological treatments do not directly target the core features of ASD, treatment of co-occurring symptoms may indirectly influence current symptom presentation and functioning (44). In particular, ADHD symptoms are associated with poorer adaptive functioning in ASD, and their treatment may have functional implications (36). Thus, unmeasured differences in medication use between the ASD and SCD groups may have influenced the observed differences in current symptom severity and adaptive functioning. Future studies incorporating treatment-related factors are needed to further clarify the functional characteristics of adolescents with ASD and SCD.

### 4.3 Conclusion

In conclusion, the distinction between ASD and SCD was most evident in lifetime autism features, less pronounced in current symptom presentation, and further attenuated in adaptive functioning during adolescence. A multidimensional approach to clinical assessment and management that considers symptom profiles alongside functional, developmental, cognitive, and clinical factors may be needed for a more accurate understanding of adolescents with ASD and SCD.

## Data Availability

The data analyzed in this study are available from the corresponding author upon reasonable request, subject to approval by Neudive Inc., the data provider. Requests to access these datasets should be directed to Y-SJ, yschoung@skku.edu.

## References

[1] American Psychiatric Association . Diagnostic and Statistical Manual of Mental Disorders, Text Revision (DSM-5-TR). Washington, DC: American Psychiatric Association Publishing (2022).

[2] Kim YS Fombonne E Koh YJ Kim SJ Cheon KA Leventhal BL . A comparison of DSM-IV pervasive developmental disorder and DSM-5 autism spectrum disorder prevalence in an epidemiologic sample. J Am Acad Child Adolesc Psychiatry. (2014) 53:500–8. doi: 10.1016/j.jaac.2013.12.021 24745950 PMC4058782

[3] Swineford LB Thurm A Baird G Wetherby AM Swedo S . Social (pragmatic) communication disorder: A research review of this new DSM-5 diagnostic category. J Neurodev Disord. (2014) 6:41. doi: 10.1186/1866-1955-6-41 25484991 PMC4258293

[4] Lord C Bishop SL . Recent advances in autism research as reflected in DSM-5 criteria for autism spectrum disorder. Annu Rev Clin Psychol. (2015) 11:53–70. doi: 10.1146/annurev-clinpsy-032814-112745 25581244

[5] Ellis Weismer S Rubenstein E Wiggins L Durkin MS . A preliminary epidemiologic study of social (pragmatic) communication disorder relative to autism spectrum disorder and developmental disability without social communication deficits. J Autism Dev Disord. (2021) 51:2686–96. doi: 10.1007/s10803-020-04737-4 33037562 PMC8032796

[6] Mandy W Wang A Lee I Skuse D . Evaluating social (pragmatic) communication disorder. J Child Psychol Psychiatry. (2017) 58:1166–75. doi: 10.1111/jcpp.12785 28741680

[7] Foley-Nicpon M Fosenburg L Wurster KG Assouline SG . Identifying high ability children with DSM-5 autism spectrum or social communication disorder: Performance on autism diagnostic instruments. J Autism Dev Disord. (2017) 47:460–71. doi: 10.1007/s10803-016-2973-4 27885547

[8] Norbury CF . Practitioner review: Social (pragmatic) communication disorder conceptualization, evidence and clinical implications. J Child Psychol Psychiatry. (2014) 55:204–16. doi: 10.1111/jcpp.12154 24117874

[9] Gibson J Adams C Lockton E Green J . Social communication disorder outside autism? A diagnostic classification approach to delineating pragmatic language impairment, high functioning autism and specific language impairment. J Child Psychol Psychiatry. (2013) 54:1186–97. doi: 10.1111/jcpp.12079 23639107

[10] Flax J Gwin C Wilson S Fradkin Y Buyske S Brzustowicz L . Social (pragmatic) communication disorder: Another name for the broad autism phenotype? Autism. (2019) 23:1982–92. doi: 10.1177/1362361318822503 30931583 PMC6773532

[11] Gabbatore I Guerrini AM Bosco FM . The fuzzy boundaries of the social (pragmatic) communication disorder (SPCD): Why the picture is still so confusing? Heliyon. (2023) 9:e19062. doi: 10.1016/j.heliyon.2023.e19062 37664706 PMC10468801

[12] Edwards H Wright S Sargeant C Cortese S Wood‐Downie H . Research review: A systematic review and meta‐analysis of sex differences in narrow constructs of restricted and repetitive behaviours and interests in autistic children, adolescents, and adults. J Child Psychol Psychiatry. (2024) 65:4–17. doi: 10.1111/jcpp.13855 37485556

[13] Ward A Boan AD Carpenter LA Bradley CC . Evaluating the rate of social (pragmatic) communication disorder in children at risk for autism spectrum disorder. Children’s Health Care. (2020) 49:425–34. doi: 10.1080/02739615.2020.180307237339054

[14] Seltzer MM Krauss MW Shattuck PT Orsmond G Swe A Lord C . The symptoms of autism spectrum disorders in adolescence and adulthood. J Autism Dev Disord. (2003) 33:565–81. doi: 10.1023/b:jadd.0000005995.02453.0b 14714927

[15] Jain D Multani KS Dodiya A Benani U Iyer A . Adaptive behavior and its differences between children with autism spectrum disorder and social communication disorder. Autism. (2025) 29:829–37. doi: 10.1177/13623613251317787 39943881

[16] Chatham CH Taylor KI Charman T Liogier D’Ardhuy X Eule E Fedele A . Adaptive behavior in autism: Minimal clinically important differences on the Vineland‐II. Autism Res. (2018) 11:270–83. doi: 10.1002/aur.1874 28941213 PMC5997920

[17] Hill TL Gray SA Kamps JL Enrique Varela R . Age and adaptive functioning in children and adolescents with ASD: The effects of intellectual functioning and ASD symptom severity. J Autism Dev Disord. (2015) 45:4074–83. doi: 10.1007/s10803-015-2522-6 26174048 PMC5087278

[18] Kanne SM Gerber AJ Quirmbach LM Sparrow SS Cicchetti DV Saulnier CA . The role of adaptive behavior in autism spectrum disorders: Implications for functional outcome. J Autism Dev Disord. (2011) 41:1007–18. doi: 10.1007/s10803-010-1126-4 21042872

[19] Lee EY Cho S Ju R Kim H Choi TY Yoo JH . Development of a social communication intervention mobile app for adolescents with autism spectrum disorder and social communication disorder: protocol for a pilot randomized clinical trial. JMIR Res Protoc. (2025) 14:e66419. doi: 10.2196/66419 40925589 PMC12457866

[20] Yoo JH Lee EY Lee JW Kim H Cho SJ Ju R . Effectiveness of a mobile application game (NDTx‐01) in enhancing social communication skills in adolescents with autism spectrum disorder or social communication disorder: A randomized controlled pilot trial. Psychiatry Clin Neurosci. (2025) 79:389–97. doi: 10.1111/pcn.13824 40214154 PMC12232077

[21] Lord C Rutter M Le Couteur A . Autism Diagnostic Interview-Revised: a revised version of a diagnostic interview for caregivers of individuals with possible pervasive developmental disorders. J Autism Dev Disord. (1994) 24:659–85. doi: 10.1007/BF02172145 7814313

[22] Yoo HJ Park K Kwak Y Cho S Ban G Kim B . Korean Version of Autism Diagnostic Interview-Revised (ADI-R). Seoul: Hakjisa (2007).

[23] Oh M Song DY Bong G Yoon NH Kim SY Kim JH . Validating the autism diagnostic interview-revised in the korean population. Psychiatry Investig. (2021) 18:196–204. doi: 10.30773/pi.2020.0337 33735550 PMC8016687

[24] Constantino JN . Social responsiveness scale. In: Encyclopedia of Autism Spectrum Disorders. Cham, Switzerland: Springer International Publishing (2021), 4457–67.

[25] Chun J Bong G Han JH Oh M Yoo HJ . Validation of social responsiveness scale for Korean preschool children with autism. Psychiatry Invest. (2021) 18:831. doi: 10.30773/pi.2021.0182 34500507 PMC8473854

[26] Bölte S Poustka F Constantino JN . Assessing autistic traits: cross‐cultural validation of the social responsiveness scale (SRS). Autism Res. (2008) 1:354–63. doi: 10.1002/aur.49 19360690

[27] Sparrow SS Cicchetti DV Balla DA . Vineland Adaptive Behavior Scales–Second Edition (Vineland–II). Circle Pines, MN: American Guidance Service (2005).

[28] Hwang S Kim J Hong S Bae S Cho S . Standardization study of the Korean vineland adaptive behavior scales-II (K-vineland-II). Korean J Clin Psychol. (2015) 34:851–76. doi: 10.15842/kjcp.2015.34.4.002

[29] Operto FF Pastorino GMG Scuoppo C Padovano C Vivenzio V Pistola I . Adaptive behavior, emotional/behavioral problems and parental stress in children with autism spectrum disorder. Front Neurosci. (2021) 15:751465. doi: 10.3389/fnins.2021.751465 34899160 PMC8660640

[30] Kwak KJ Oh SW Kim CT . Korean-Wechsler Intelligence Scale for Children. Seoul: Hakjisa (2011) p. 11–3.

[31] Tillmann J San José Cáceres A Chatham CH Crawley D Holt R Oakley B . Investigating the factors underlying adaptive functioning in autism in the EU‐AIMS Longitudinal European Autism Project. Autism Res. (2019) 12:645–57. doi: 10.1002/aur.2081 30741482 PMC6519242

[32] Bottini S . Social reward processing in individuals with autism spectrum disorder: A systematic review of the social motivation hypothesis. Res Autism Spectr Disord. (2018) 45:9–26. doi: 10.1016/j.rasd.2017.10.00142574925

[33] Lindsay‐Webb K Clayton P Simonoff E Hollocks MJ . Examining the relationship between social motivation and internalizing symptoms in autistic people: A systematic review and meta‐Analysis. Autism Res. (2026) 19:e70185. doi: 10.1002/aur.70185 41670017 PMC12996855

[34] Briot K Jean F Jouni A Geoffray MM Ly-Le Moal M Umbricht D . Social anxiety in children and adolescents with autism spectrum disorders contribute to impairments in social communication and social motivation. Front Psychiatry. (2020) 11:710. doi: 10.3389/fpsyt.2020.00710 32793002 PMC7393242

[35] Szatmari P Georgiades S Duku E Bennett TA Bryson S Fombonne E . Developmental trajectories of symptom severity and adaptive functioning in an inception cohort of preschool children with autism spectrum disorder. JAMA Psychiatry. (2015) 72:276–83. doi: 10.1001/jamapsychiatry.2014.2463 25629657

[36] Chandler S Carter Leno V White P Yorke I Hollocks MJ Baird G . Pathways to adaptive functioning in autism from early childhood to adolescence. Autism Res. (2022) 15:1883–93. doi: 10.1002/aur.2785 35899846 PMC9796413

[37] Pugliese CE Anthony L Strang JF Dudley K Wallace GL Kenworthy L . Increasing adaptive behavior skill deficits from childhood to adolescence in autism spectrum disorder: Role of executive function. J Autism Dev Disord. (2015) 45:1579–87. doi: 10.1007/s10803-014-2309-1 25398602 PMC4433442

[38] Redmond SM . Clinical intersections among idiopathic language disorder, social (pragmatic) communication disorder, and attention-deficit/hyperactivity disorder. J Speech Language Hearing Res. (2020) 63:3263–76. doi: 10.1044/2020_jslhr-20-00050 33064599 PMC8363244

[39] Klin A Saulnier CA Sparrow SS Cicchetti DV Volkmar FR Lord C . Social and communication abilities and disabilities in higher functioning individuals with autism spectrum disorders: The Vineland and the ADOS. J Autism Dev Disord. (2007) 37:748–59. doi: 10.1007/s10803-006-0229-4 17146708

[40] Jin WY Song C Wang YY Liu XL Li WH Wu LL . Effects of intelligence levels and autistic severity on adaptive functioning and cognitive-adaptive functioning gaps in school-aged children with autism spectrum disorder. Neuropsychiatr Dis Treat. (2025) 21:1131–42. doi: 10.2147/NDT.S524042 40492028 PMC12146886

[41] McQuaid GA Pelphrey KA Bookheimer SY Dapretto M Webb SJ Bernier RA . The gap between IQ and adaptive functioning in autism spectrum disorder: Disentangling diagnostic and sex differences. Autism. (2021) 25:1565–79. doi: 10.1177/1362361321995620 33715473 PMC8324508

[42] Miranda A Berenguer C Baixauli I Roselló B . Childhood language skills as predictors of social, adaptive and behavior outcomes of adolescents with autism spectrum disorder. Res Autism Spectr Disord. (2023) 103:102143. doi: 10.1016/j.rasd.2023.10214342574925

[43] Wan E Penner M Crosbie J Brian J Kelley E Schachar R . Transdiagnostic behavioral and sociodemographic influences on the cognitive-adaptive functioning gap in neurodivergent children. Sci Rep. (2026). doi: 10.1038/s41598-026-58625-5 42431939 PMC13654804

[44] Persico AM Ricciardello A Lamberti M Turriziani L Cucinotta F Brogna C . The pediatric psychopharmacology of autism spectrum disorder: A systematic review-Part I: The past and the present. Prog Neuro-Psychopharmacol Biol Psychiatry. (2021) 110:110326. doi: 10.1016/j.pnpbp.2021.110326 33857522

