# Supplementary material for: Differences in autism features and adaptive functioning between adolescents with autism spectrum disorder and social communication disorder
Source: Front Psychiatry. 2026 Oct 2;17:1961001. doi: 10.3389/fpsyt.2026.1961001 (PMC13672609; doi:10.3389/fpsyt.2026.1961001)
Supplement: Supplementary file 1 [file Table1.docx]

Supplementary Material

# Table S1. Type III Interaction Tests and BH-FDR Adjusted *p*-values for Moderator × Group Effects Across ADI-R, SRS-2, and VABS-II

| **ADI-R domain** | | **A: Abnormalities in Reciprocal social interaction** | | | | | | **B: Abnormalities in Communication** | | | | | | | | **C: RRB** | | | | | | | **D: Abnormality of Development evident  at or before 36 months** | | | | | |
| --- | --- | --- | --- | --- | --- | --- | --- | --- | --- | --- | --- | --- | --- | --- | --- | --- | --- | --- | --- | --- | --- | --- | --- | --- | --- | --- | --- | --- |
| **Moderator** | | ***p*** | | | **Adj. *p*** | | | ***p*** | | | | **Adj. *p*** | | | | ***p*** | | | **Adj. *p*** | | | | ***p*** | | | | **Adj. *p*** | |
| Age | | .321 | | | .855 | | | .471 | | | | .855 | | | | .247 | | | .768 | | | | .983 | | | | .983 | |
| Sex | | .438 | | | .855 | | | .137 | | | | .548 | | | | .862 | | | .951 | | | | .577 | | | | .855 | |
| FSIQ | | .351 | | | .855 | | | .197 | | | | .690 | | | | .481 | | | .855 | | | | .811 | | | | .951 | |
| Psychiatric comorbidity | | .130 | | | .548 | | | .087 | | | | .548 | | | | .118 | | | .548 | | | | .501 | | | | .855 | |
| ADHD | | .086 | | | .548 | | | .120 | | | | .548 | | | | .061 | | | .548 | | | | .560 | | | | .855 | |
| Lifetime Tx | | .375 | | | .855 | | | .641 | | | | .855 | | | | .910 | | | .951 | | | | .595 | | | | .855 | |
| Ongoing Tx | | .771 | | | .951 | | | .830 | | | | .951 | | | | .611 | | | .855 | | | | .917 | | | | .951 | |
| **SRS-2 Subscale** | **Social Awareness** | | | | | **Social Cognition** | | | | **Social Communication** | | | | **Social Motivation** | | | | **RRB** | | | | **Social Communication Index** | | | | **Total** | | |
| **Moderator** | ***p*** | | **Adj. *p*** | ***p*** | | | **Adj. *p*** | | ***p*** | | **Adj. *p*** | | ***p*** | | **Adj. *p*** | | ***p*** | | | **Adj. *p*** | ***p*** | | | **Adj. *p*** | ***p*** | | | **Adj. *p*** |
| Age | .520 | | .979 | .872 | | | .979 | | .696 | | .979 | | .513 | | .979 | | .535 | | | .979 | .835 | | | .979 | .973 | | | .979 |
| Sex | .888 | | .979 | .655 | | | .979 | | .827 | | .979 | | .516 | | .979 | | .973 | | | .979 | .518 | | | .979 | .621 | | | .979 |
| FSIQ | .445 | | .979 | .669 | | | .979 | | .409 | | .979 | | .436 | | .979 | | .677 | | | .979 | .338 | | | .979 | .431 | | | .979 |
| Psychiatric comorbidity | .979 | | .979 | .933 | | | .979 | | .138 | | .979 | | .137 | | .979 | | .543 | | | .979 | .315 | | | .979 | .479 | | | .979 |
| ADHD | .547 | | .979 | .694 | | | .979 | | .312 | | .979 | | .755 | | .979 | | .865 | | | .979 | .454 | | | .979 | .577 | | | .979 |
| Lifetime Tx | .281 | | .979 | .219 | | | .979 | | .909 | | .979 | | .938 | | .979 | | .802 | | | .979 | .819 | | | .979 | .883 | | | .979 |
| Ongoing Tx | .655 | | .979 | .496 | | | .979 | | .919 | | .979 | | .867 | | .979 | | .735 | | | .979 | .941 | | | .979 | .945 | | | .979 |

| **VABS-II domain** | **Communication** | | **Daily living** | | **Socialization** | | **ABC** | |
| --- | --- | --- | --- | --- | --- | --- | --- | --- |
| **Moderator** | ***p*** | **Adj. *p*** | ***p*** | **Adj. *p*** | ***p*** | **Adj. *p*** | ***p*** | **Adj. *p*** |
| Age | .196 | .457 | .663 | .787 | .903 | .915 | .444 | .720 |
| Sex | .509 | .750 | .702 | .787 | .592 | .753 | .703 | .787 |
| FSIQ | .915 | .915 | .148 | .414 | .564 | .752 | .413 | .720 |
| Psychiatric comorbidity | **.003*** | **.047*** | .117 | .410 | **.005*** | **.047*** | **.004*** | **.047*** |
| ADHD | **.046*** | .322 | .874 | .915 | .114 | .410 | .146 | .414 |
| Lifetime Tx | .092 | .410 | .226 | .487 | .168 | .428 | .116 | .410 |
| Ongoing Tx | .305 | .610 | .463 | .720 | .538 | .752 | .436 | .720 |

**Note.** *p*-values represent Type III tests of the Moderator × Group interaction term. Adj. *p*-values were calculated using the Benjamini–Hochberg false discovery rate (BH-FDR) correction within each measure. ADI-R: Autism Diagnostic Interview–Revised; SRS-2: Social Responsiveness Scale, Second Edition; VABS-II: Vineland Adaptive Behavior Scales, Second Edition; RRB: Restricted and Repetitive Behaviors; ABC: Adaptive Behavior Composite; FSIQ: Full-scale Intelligence Quotient; ADHD: Attention-Deficit/Hyperactivity Disorder; Lifetime Tx: Lifetime treatment history; Ongoing Tx: Ongoing treatment status. *** *p* < .05**
